# Volcanic suppression of Nile summer flooding triggers revolt and constrains interstate conflict in ancient Egypt

**DOI:** 10.1038/s41467-017-00957-y

**Published:** 2017-10-17

**Authors:** Joseph G. Manning, Francis Ludlow, Alexander R. Stine, William R. Boos, Michael Sigl, Jennifer R. Marlon

**Affiliations:** 1 Departments of History and Classics, Yale University, New Haven, CT 06520 USA; 2 Yale Law School, New Haven, CT 06511 USA; 3 Yale Climate & Energy Institute and Department of History, Yale University, New Haven, CT 06511 USA; 4 Department of History, School of Histories & Humanities, Trinity College, Dublin 2, Ireland; 5 Department of Earth & Climate Sciences, San Francisco State University, San Francisco, CA 94132 USA; 6 Department of Earth and Planetary Science, University of California, Berkeley, CA 94720 USA; 7 Climate and Ecosystem Sciences Division, Lawrence Berkeley National Laboratory, Berkeley, CA 94720 USA; 8 Laboratory of Environmental Chemistry, Paul Scherrer Institute, 5232 Villigen, Switzerland; 9 Oeschger Centre for Climate Change Research, University of Bern, Bern, 3012 Switzerland; 10 School of Forestry & Environmental Studies, Yale University, New Haven, CT 06511 USA

## Abstract

Volcanic eruptions provide tests of human and natural system sensitivity to abrupt shocks because their repeated occurrence allows the identification of systematic relationships in the presence of random variability. Here we show a suppression of Nile summer flooding via the radiative and dynamical impacts of explosive volcanism on the African monsoon, using climate model output, ice-core-based volcanic forcing data, Nilometer measurements, and ancient Egyptian writings. We then examine the response of Ptolemaic Egypt (305–30 BCE), one of the best-documented ancient superpowers, to volcanically induced Nile suppression. Eruptions are associated with revolt onset against elite rule, and the cessation of Ptolemaic state warfare with their great rival, the Seleukid Empire. Eruptions are also followed by socioeconomic stress with increased hereditary land sales, and the issuance of priestly decrees to reinforce elite authority. Ptolemaic vulnerability to volcanic eruptions offers a caution for all monsoon-dependent agricultural regions, presently including 70% of world population.

## Abstract (web summary)

The degree to which human societies have responded to past climatic changes remains unclear. Here, using a novel combination of approaches, the authors show how volcanically-induced suppression of Nile summer flooding led to societal unrest in Ptolemaic Egypt (305–30 BCE).

## Introduction

**Fig. 1:**
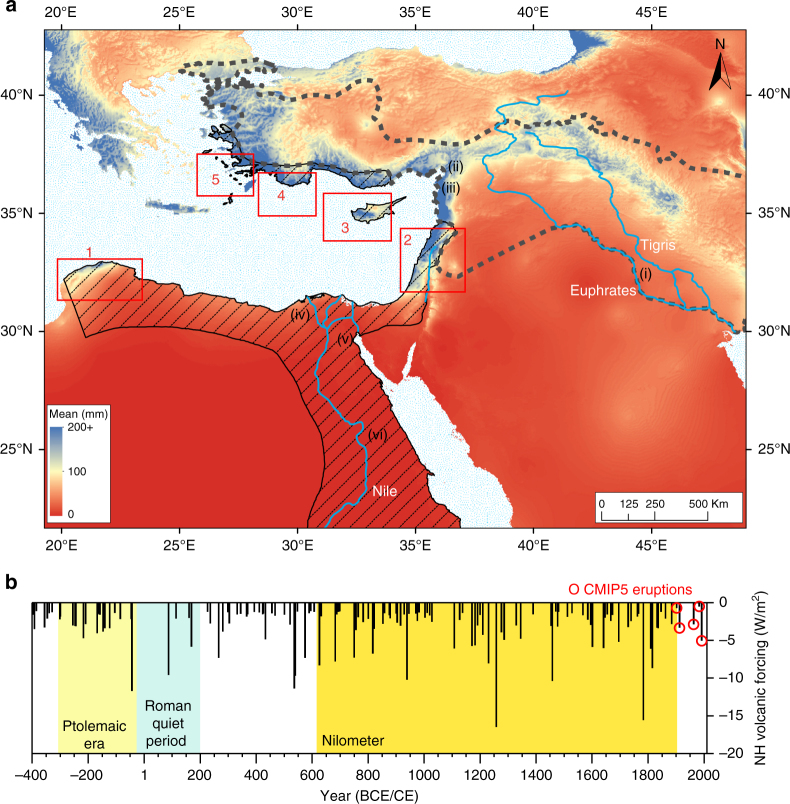
Political and environmental setting with volcanic forcing history. **a** Eastern Mediterranean wet season rainfall (taking as example the December mean, mm, 1950–2000, at 30 arc-seconds resolution, worldclim.org, v1.4)^66^, indicative of Ptolemaic and Seleukid territories potentially capable of rainfed agriculture, with state boundaries c.275 BCE overlain: Ptolemaic (black line with diagonal dashes) and Seleukid (thick gray dashes)^67^. Key territories contested by these states are numbered with red Arabic numerals alongside indicative rectangles (1, Cyrenaica; 2, ‘Koile’ Syria; 3, Cyprus; 4, Lycia; 5, Caria) and are observed to focus on regions potentially capable of rainfed agriculture. Selected urban power bases are located with Roman numerals ((i), Babylon; (ii), Antioch; (iii), Seleukia; (iv), Alexandria; (v), Memphis; (vi), Thebes). **b** Ice-core-indicated dates of maximum aerosol forcing from volcanic eruptions with time-integrated (i.e., cumulative (Methods section)) forcing estimates for the Northern Hemisphere, 400 BCE to the present^27^. CMIP5 eruptions a﻿re those five twentieth century eruptions included in the Coupled Model Intercomparison Project Phase 5 (Methods)

The need to adapt to and mitigate the impacts of anthropogenic climate change has revived interest in longstanding but unsettled questions concerning how past climatic changes have influenced human societies^1^. Egypt provides a unique historical laboratory in which to study social vulnerability and response to abrupt hydroclimatic shocks. As one of the Ancient World’s great “hydraulic civilizations”^2^, its prosperity was overwhelmingly tied to the annual cycle of Nile summer flooding, with diminished flooding (Nile failure) often associated with major human impacts through its many millennia of recorded history^3^. Of all Ancient Egyptian history, that of Ptolemaic Egypt (305–30 BCE; Fig. 1a) is most richly furnished with contemporary documentation. As the longest-lived successor to Alexander the Great’s empire, the Ptolemaic state was a major force in the transformative Hellenistic era, a period marked by large-scale conflict but also material and cultural achievement. Ptolemaic Egypt featured one of the largest cities of the Ancient Mediterranean (Alexandria), including the Great Library and Lighthouse, and was a hub for invention, boasting minds such as Euclid and Archimedes. Technological advances such as the *saqiya* ^4^, a rotary-wheel water-lifting machine documented by the mid-third century BCE, maslin (mixed wheat-barley) cropping, as well as grain storage, acted to mitigate the impacts of the mercurial Nile flood (Supplementary Note 1)^5^. Families also distributed land in geographically dispersed individual shares to further hedge against the risk of Nile failure, and tailored agricultural decisions to annual flood conditions^6^. External territories (e.g., Anatolia, Syria; Fig. 1a) capable of rainfed agriculture also helped buffer the state against Nile failure (Supplementary Note 2)^7^. The existence of these mitigation and adaptation strategies highlights the importance of managing Nile variability in Ptolemaic Egypt, yet discussion of the impact of hydroclimatic shocks is effectively absent from modern histories of the period.

At ~6825 km, the Nile is among the Earth’s great rivers, fed by rainfall in Africa’s equatorial plateau (mainly via the White Nile) and the Ethiopian Highlands (mainly via the Blue Nile and Atbara rivers)^8^. Before twentieth century damming, the summer flood, driven primarily by monsoon rainfall in the Ethiopian highlands, began with rising waters observed at Aswan as early as June, peaking from August to September, and largely receding by the end of October, when crop sowing began^2^. Nile flood suppression from historical eruptions has been little studied, despite Nile failures with severe social impacts coinciding with eruptions such as Eldgjá, c.939, Laki, 1783–1784, and Katmai, 1912^9, 10^.

Explosive eruptions can perturb climate by injecting sulfurous gases into the stratosphere; these gases react to form reflective sulfate aerosols that remain aloft in decreasing concentrations for approximately one to two years^11^. While most studies of the climatic effects of volcanism have focused on temperature changes, contemporary and historical societies were also vulnerable to hydrological changes^12^. Hydroclimate is harder to reconstruct and model, but studies are increasingly noting global and regional hydroclimatic impacts from explosive volcanism^10, 13–20^. Volcanic aerosols influence hydroclimate through multiple mechanisms. Aerosol scattering of solar radiation to space reduces tropospheric temperatures; if lower-tropospheric relative humidities remain unchanged, the mass of water converged by a given wind distribution decreases, and precipitation minus surface evaporation (P-E) is thus reduced^21^. This thermodynamic effect may represent the principal means by which equatorially symmetric aerosol distributions from tropical eruptions alter P–E^15^. In addition, extratropical eruptions increase sulfate aerosols on one side of the equator, cool that hemisphere, and may thus alter tropical P–E primarily by changing winds. In particular, a high-latitude energy sink in one hemisphere forces an anomalous Hadley circulation, shifting the intertropical convergence zone (ITCZ) away from that energy sink^16, 22^. Aerosol cooling of northern high latitudes can thus force a southward shift of northern hemisphere (NH) summer monsoon precipitation, promoting drought in the northern parts of monsoon regions^16–18, 23^. These energy-budget arguments provide a more fundamental perspective on the controls on tropical rainfall than arguments based on land-ocean temperature contrast because large-scale tropical circulations are driven by horizontal gradients in the total (sensible plus latent) energy input to the atmosphere^24^. The hypothesis that a decrease in land-ocean temperature contrast will cause monsoon rainfall to weaken has been disproven by the observation that continental monsoon regions are cooler during years of enhanced monsoon precipitation^25^, and by the fact that monsoon winds weaken as land-ocean temperature contrast strengthens in projections of next-century warming^26^.

In what follows, modeling of twentieth century explosive eruptions, together with newly revised ice-core records of volcanic climate forcing^27^ and the Islamic Nilometer (622–1902 CE)^28^, allow us to establish the Nile’s physical response to explosive volcanism. We replicate this response in earlier centuries using Ptolemaic era flood quality descriptions^29^, and further employ the period’s abundant documentary record to examine the role of repeated volcanic flood suppression in Ptolemaic political and social history.

## Results

### Climate model output

**Fig. 2:**
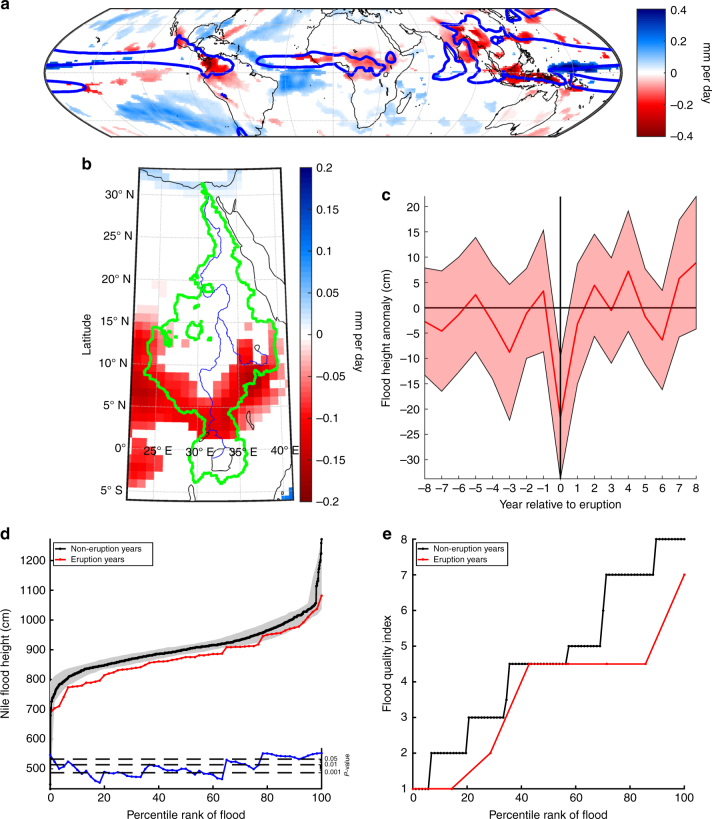
Response of the Nile to explosive volcanism. **a** CMIP5 ensemble mean precipitation minus evaporation (P–E) response to five 20th century volcanic eruptions (color shading, mm per day). The response is the average P–E over the first summer season (May–October) that contained or followed the eruption, relative to the five summers preceding the eruption. Only anomalies statistically significant at the 5% level are shown (Methods section). Blue contour is the 2 mm per day isoline of P–E. **b** As in **a** but showing the Nile watershed, which is outlined in green, with major Nile watercourses shown by thin blue lines. **c** Annual Nile summer flood heights from the Islamic Nilometer composited relative to the ice-core-estimated dates of eruption years for 60 large eruptions (Methods section) between 622 and 1902 CE (eruption years are represented at point 0 on the horizontal axis; years 1 to 8 then represent the first to eighth years after these eruptions, and years −1 to −8 the first to eighth years before). Data associated with secondary eruptions within this compositing window are excluded (Methods section). Shading indicates the 2-tailed 90% confidence interval, estimated using the *t*-distribution. Nile summer flood heights average 22 cm lower in eruption years (*P* < 0.01). **d** Annual Nile summer flood heights from the Islamic Nilometer ranked by percentile for eruption years (red; *N* = 61 (Methods section)) and non-eruption years (black; *N* = 1030). Gray shading indicates the 2-tailed Monte Carlo 90% confidence interval based on 1,000,000 draws from the non-eruption-year distribution. The blue line indicates the 1-tailed *P*-value for each of 61 ranks, when tested independently without accounting for multiple tests. **e** Annual Nile flood quality index for the Ptolemaic period, 305–30 BCE (Methods section)^29^, ordered by percentile for eruption years (red; *N* = 8) and non-eruption years (black; *N* = 88). High flood quality index values equate to documentary indications of high summer floods

Observations and model simulations indicate that, after five twentieth century eruptions (Fig. 1b), precipitation was suppressed across the Sahel into Ethiopia and in the equatorial regions of Africa that feed the White and Blue Niles^19^. An ensemble of climate models (Methods section) forced by these eruptions (i.e., CMIP5 eruptions, Fig. 1b) shows a reduction in P-E (i.e., net water available for runoff) in NH monsoon regions during boreal summer (Fig. 2a). This results primarily from a weakening (rather than a shift) of the ITCZ, consistent with only one of these eruptions being extratropical (Katmai/Novarupta, Alaska, June 1912). A southward ITCZ shift is evident in the West Pacific and East Atlantic, and the response to individual eruptions may show larger shifts than the ensemble mean due to meridional asymmetries in the radiative forcing produced even by tropical eruptions^13^. Nevertheless, the ensemble mean P-E anomaly in central and eastern Africa shows no dipole structure indicative of a meridional ITCZ shift. Volcanically induced drying occurs primarily in the southern Nile watershed, and the P-E signal indicates that volcanically induced cooling did not sufficiently inhibit evaporation in the watershed to compensate for decreased rainfall (Fig. 2b). Integrated over the watershed, the simulated P-E reduction is only about 5% of the climatological mean, but observations show stronger drying than the models^19^; stronger drying may also occur after bigger tropical eruptions or extratropical eruptions inducing an ITCZ shift. Moreover, the largest five eruptions of the 20th century (examined in the above modeling) rank on average only 132nd among the 283 largest volcanic perturbations since 500 BCE^27^, thereby undersampling the variability of historical volcanic aerosol climate forcing, and motivating our study of historical volcanism using ice-core-based volcanic forcing reconstructions with long temporal span (i.e., Fig. 1b).

### Historical flood height analyses

To thus examine the influence of historical volcanism, we employ summer flood measurements from the Islamic Nilometer, the longest-known annually recorded observational hydrological record, starting 622 CE^28^. We composite annual summer flood height values up to 1902 CE, when the Aswan Low Dam was completed, relative to eruptions with reconstructed NH forcing of <−1 W/m^2^ (Figs. 1b, 2c, Methods section)^27^. Eruption-year summer flooding averaged 22 cm lower than non-eruption years, statistically significant at *P* < 0.01 (1-tail, 2-sample *t*-test). To further establish the association between eruptions and Nile summer flood heights, we compare ranked flood heights for eruption vs. non-eruption years as a function of ranked percentile (Fig. 2d). We find that eruption-year summer flood height is consistently lower than the equivalent non-eruption years in all but one case (the 0th percentile (which we define as the minimum value of the set)). Monte Carlo analysis indicates that, excepting the 0th percentile, the eruption-year distribution is statistically significantly lower than the non-eruption year distribution for all heights below the 78th percentile (*P* < 0.05, Fig. 2d). These results support our understanding of volcanic impacts on the summer flood. Additionally, while the interaction between eruption size and the scale of Nile summer flood suppression is complex, assessment of the sensitivity of our result suggests that eruptions with larger NH forcing produce a greater suppression of Nile summer flood heights (Methods section).

The great socioeconomic importance of the Nile summer flood ensured its frequent mention (directly and indirectly) in ancient writings before the 622 CE start of the Islamic Nilometer data. Using these writings, qualitative flood quality indications can be placed on an ordinal scale for 96 years in the Ptolemaic period, 305–30 BCE (Methods section)^29^. Ranked comparison of flood quality from eruption and non-eruption years shows that eruption years exhibit the same or lower flood quality than non-eruption years for all percentiles (Fig. 2e), consistent with an expected volcanic reduction of the summer flood in this pre-Islamic-Nilometer era. Reconstructions based on documentary records are, however, vulnerable to the dating uncertainties (Methods section). To account for this risk, we further compare Nile flood quality for years within one year of an eruption to all other years and find that flood quality is significantly lower close in time to eruptions (*P* < 0.04, 1-tail 2-sample *t*-test), again indicating the persistence of volcanic impacts on the Nile in the Ptolemaic period.

### Societal response

For the first time in Egyptian history, well-dated records, especially papyri, survive in large numbers during the Ptolemaic period. In tandem with newly revised and extended ice-core volcanic forcing data^27^, these allow us to compile indexes of socioeconomic and political activity (Methods section) to identify potential top-down (state/elite level) and bottom-up responses to volcanically induced hydrological shocks. We thus examine the timing and frequency of Ptolemaic interstate warfare and the issuance of priestly decrees (primarily top–down responses) relative to eruption years, as well as the timing and frequency of revolt onset against Ptolemaic rule and hereditary land sales (primarily bottom-up responses).

**Fig. 3:**
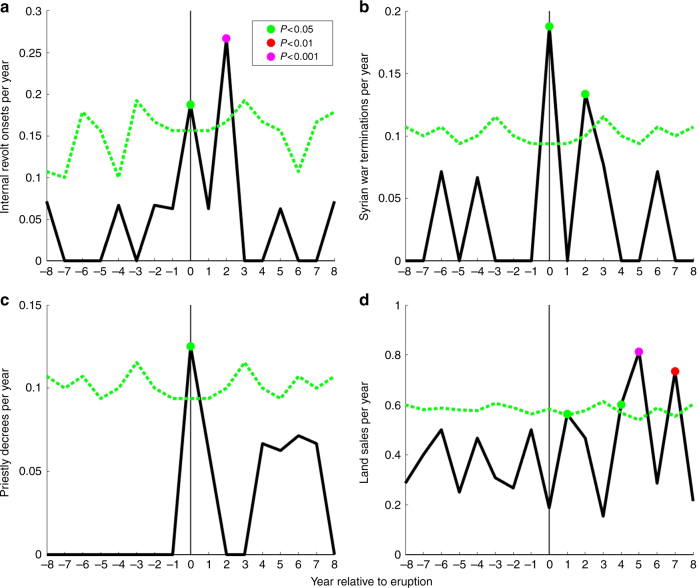
Response to volcanism in Egyptian social indexes. **a** Dates of the initiation of internal revolts against Ptolemaic rule composited relative to the dates of 16 volcanic eruptions (represented at year 0 on the horizontal axes; years 1–8 then represent the first to eighth years after these eruptions, and years −1 to −8 the first to eighth years before), excluding an 8-year buffer at the start and end of our 305–30 BCE period (Methods section). Dots indicates statistically significant values estimated using Barnard’s exact test (green: *P* < 0.05, red: *P* < 0.01, magenta: *P* < 0.001). Green dashed lines give the 95% confidence threshold also estimated using Barnard’s exact test. Data associated with any secondary eruptions within the compositing window are excluded (Methods section), and remaining data expressed as a fraction of all records to prevent bias. **b** Same as **a**, but for the dates of terminations of the “Syrian Wars”, i.e., wars between the Ptolemaic and Seleukid states. **c** Same as **a**, but for priestly decrees. **d** Same as **a**, but for Demotic language land sales with significance estimated using the 2-sample student’s *t*-test. Revolt onset dates *N* = 10; Syrian Wars *N* = 9; decrees *N* = 9; land sales *N* = 84. Eruption years *N* = 18, and non-eruption years *N* = 258 (305–30 BCE)

Revolts of varying severity and extent are signaled in papyri and inscriptions, with an absence of tax receipts also indicating significant loss of state control^30^, as during the great 20-year Theban revolt starting 207 BCE (Methods section; Supplementary Table 2). These revolts are generally considered “nationalist” uprisings by Egyptians resentful of Greek (i.e., Ptolemaic) rule, and/or burdensome state taxation^30^, and are rarely considered in relation to socioeconomic stresses following Nile failure^31^. To examine this further, we composite revolt onset dates relative to eruption years and observe an increase in revolt onset frequency in these years (*P* < 0.05, Barnard’s exact test, Fig. 3a, Methods section), implicating volcanically induced Nile failure as an additional and hitherto little recognized potential catalyst for revolt against Ptolemaic rule. A prominent peak in the second years following eruptions (*P* < 0.001) also suggests a multi-year or lagged response, with revolt onset in some instances plausibly delayed or potentially prevented by short-term coping strategies such as Cleopatra’s release of state-reserved grain during documented Nile failure after eruptions in 46 and 44 BCE (Supplementary Note 3)^32^.

The Ptolemies fought nine “Syrian wars” with their great Near Eastern rival, the Seleukid Empire (Fig. 1a), between 274 BCE and 96 BCE (Supplementary Table 3)^33^, involving some of the largest military mobilizations in ancient Mediterranean history. The observed association between eruptions and internal revolt onset motivates us to examine whether eruptions are a potential influence on interstate conflict. We find no significant association when compositing war initiation dates relative to eruptions (not shown), suggesting initiation is driven mainly by other factors such as succession disputes^33^. Mapping contested territories against regional rainfall patterns suggests that Ptolemaic access to Nile-independent rainfed territories was one additional strategic motivation (Fig. 1a). Compositing war cessation dates relative to eruptions reveals, by contrast, a statistically significant incidence in eruption years and in the second years after (*P* < 0.05, Barnard’s exact test, Fig. 3b), implicating volcanically induced Nile failure as a constraint on interstate warfare.

Historical sources are helpful in examining how this may occur, here in the context of major eruptions in 247 and 244 BCE^27^. In 245 BCE, during the third war, the surviving writings of Roman historian Justin thus note that if Ptolemy III “had not been recalled to Egypt by disturbances at home, [he] would have made himself master of all Seleucus’s dominions”, having reached as far east as Babylon on the Euphrates in a highly successful campaign (Supplementary Notes 4 and 5)^33^. A third century BCE papyrus corroborates Ptolemy’s recall to face “Egyptian revolt” at this time, while the priestly Canopus decree of 238 BCE explicitly refers to Nile failure in the preceding years and continues by commending Ptolemy III for extensive grain importation from external rainfed territories, an undertaking achieved only “at great expense” by having “sacrificed a large part of their [i.e., the dynasty’s] revenues for the salvation of the population” (Supplementary Note 2)^34, 35^. The need to quell domestic unrest as well as manage and finance large-scale relief efforts (rather than expensive military campaigning) thus plausibly contributed to the third war’s ultimate cessation by 241 BCE, providing a suggestive case of how eruption-related Nile failure may have more broadly acted to constrain interstate warfare.

Priestly decrees such as the Canopus Decree, or the Memphis Decree as famously written on the trilingual Rosetta Stone (Supplementary Table 4), praise kings in theological terms and have been interpreted as reflecting political accommodations with the influential priestly class to maintain and reinforce state authority^36^. We thus hypothesize that decrees acted as instruments by the Ptolemaic ruling elite to re-affirm control during crises. Compositing decrees relative to eruptions reveals a statistically significant incidence in eruption years (*P* < 0.05, Barnard’s exact test; Fig. 3c). We interpret this hitherto unobserved association as indicative of the functioning of decrees in reinforcing and legitimizing state rule through religious sanction during and following volcanically induced Nile failure.

Private ownership with intergenerational transfer of land within families was foundational to customary Egyptian landholding and, because all land carried tax liability, state revenues^37^. Extra-familial land sales have been posited to occur partly in response to socioeconomic stress^37^. To thus test whether eruptions prompted land sales, we composite sales and find a significant increase in their post-eruption frequency (*P* < 0.05, 2-sample *t*-test; Fig. 3d) in years 1, 4, 5, and 7. This suggests a multi-year response to volcanically induced Nile failure by families potentially forced to sell land after low harvest yields and associated difficulties meeting state taxes and other obligations. We also note that state intervention through auctioned land sales is first evidenced in the late third century BCE^38^, particularly associated with the great Theban revolt (starting 207 BCE (Supplementary Table 2)) that followed a 209 BCE tropical eruption^27^. One papyrus reports that “at the time of the revolt… most of the farmers were killed and the land has gone dry… When… [this]… was registered among the “ownerless land,”… survivors encroached upon the land… Their names are unknown since nobody pays taxes for this land to the treasury…” (Supplementary Note 6)^37^. Our results also suggest that state intervention in land auctions represent a further state-level coping strategy to return land to taxable production after Nile failure and related social instability.

## Discussion

Our results demonstrate a systematic Nile flood suppression from historical eruptions using the multi-century Islamic Nilometer and earlier written records; we find this suppression consistent with theory and modeling of volcanic monsoon impacts. We further identify statistically significant associations between eruptions and the onset of hitherto poorly understood revolts in Ptolemaic Egypt, as well as the cessation dates of Ptolemaic interstate warfare with the great Near Eastern Seleukid Empire. We stress that care must be taken to avoid engaging in and propagating a recently resurgent environmental determinism when interpreting or assigning causality (particularly monocausality) to associations observed between environmental and societal phenomena^39^. Theoretical and empirical frameworks illustrate the intricacy of coupled human-natural (or socioecological) systems, in which an interplay of influences are seen to contribute to any given societal response to a climatic or environmental shock^40–45^. We thus interpret our results as identifying a role for volcanically induced Nile failure as a trigger for revolt in Ptolemaic Egypt, and a constraint on Ptolemaic interstate conflict, against a background of multiple interacting and enabling societal stressors, or primers. These include ethnic tensions between Egyptians and Greek elites^30, 31, 35^, growing demographic and fiscal pressures^46^, burdensome state taxation^30^, the mounting costs of large semi-permanent military mobilizations^47^, and increasing urban and export demand for drought-vulnerable free-threshing wheat^48^. We similarly interpret a correspondence between priestly decrees and eruptions as a state response to related instability, and interpret increased extra-familial land sales as symptomatic of multi-year socioeconomic stress from volcanically induced Nile failure.

Our results advance the understanding of Ptolemaic state and societal responses to hydrological shocks. These now warrant consideration for their potential contribution to the state’s declining political stability after the successful reigns of Ptolemy I to III (305–222 BCE; Supplementary Table 3)^49, 50^, within the context of the political and socioeconomic pressures outlined above, alongside heavy losses of external Nile-independent rainfed territory post-195 BCE^32^. The volcanically perturbed 160’s BCE (eruptions in 168, 164, 161 BCE^27^), which contrast markedly with the quiescent “Roman optimum” of the first two centuries CE (Fig. 1b)^27, 51^, marked a decisive downturn in Ptolemaic fortunes, with two invasions by Seleukid king Antiochus IV. Only self-interested Roman intervention prevented Seleukid domination at this time. While the Ptolemaic dynasty officially ended with Cleopatra’s suicide in 30 BCE, after her naval defeat by Rome at Actium in 31 BCE^52^, it is notable that the state had been greatly impacted at the close of the preceding decade by repeated Nile failure, famine, plague, inflation, administrative corruption, rural depopulation, migration, and land abandonment (Supplementary Notes 7, 8)^32, 52^. This decade experienced the third largest eruption of the past 2500 years in 44 BCE (heavily asymmetric forcing with 87% of the aerosols remaining in the NH)^27^, closely following a 46 BCE extratropical NH eruption^27^.

The Ptolemaic era’s rich contemporary documentation, as well as the period’s archaeological potential and almost complete agricultural dependence upon the Nile summer flood (Supplementary Note 9), presents an ideal historical laboratory for further developing nuanced understandings of human-environmental relations. Ptolemaic history also highlights the urgency of considering future eruptions in risk assessment and planning for monsoon-dependent agricultural regions, comprising approximately 70% of global population^53^. Explosive eruptions are also likely to test the stability of Nile basin water usage agreements that govern and influence irrigation, hydropower, biodiversity and tourism potentials in Ethiopia, Sudan and Egypt, particularly given projected increases in Nile flow variability under future climate change^54^, and the contentious ongoing construction of the largest dam in Africa, the Grand Ethiopian Renaissance Dam on the Blue Nile^55, 56^.

## Methods

### CMIP5 analysis

Our analysis uses data from 21 models (Supplementary Table 1), many of which supply more than one integration, yielding a total of 83 model integrations that are each given equal weight in the analysis, with all data first interpolated to a standard 1° × 1° grid. The eruptions took place in October 1902 (Santa Maria, Guatemala), June 1912 (Novarupta, Alaska), March 1963 (Agung, Indonesia), March 1982 (El Chichón, Mexico), and June 1991 (Pinatubo, Philippines) (Fig. 1b). The 1912 eruption is the only extratropical eruption, so the CMIP5 model response is likely more representative of the response to tropical eruptions. The P-E response is computed by averaging over May–October of 1903, 1912, 1963, 1982, and 1991, then subtracting P–E averaged over May–October of the 5 year periods preceding each of these years. CMIP5 P–E output was provided by R. Seager via the Lamont-Doherty Earth Observatory website, as compiled for the analysis by^57^. We assess statistical significance through a Monte Carlo analysis in which 1000 synthetic series of five eruptions occurring at random years are used to create the same ensemble mean response (with averages again taken over all 83 model integrations). Each spatial grid point of the response to the true eruptions is considered statistically significant only if it is either less than the 2.5th percentile or greater than the 97.5th percentile of the distribution of synthetic responses at the same grid point. Similar analyses of CMIP5 data were conducted by^19^ and^58^.

### Social index time series

Information in all documents (inscriptions, papyri) are assessed for general reliability and authenticity through historical source and textual criticism. We focus on well-dated documents, distinguishing between the date of a text and the (potentially earlier) events described therein. To convert ancient dates in (and of) documents to the Gregorian calendar, we apply Egyptian civil calendar conversions at Frank Grieshaber, Date convertor for Ancient Egypt, http://aegyptologie.online-resourcen.de. Social unrest for Egypt is documented in diverse Greek and Egyptian (Demotic) papyri and inscriptions. We draw from the chronology of revolts reconstructed by A.E. Veïsse (Supplementary Table 2)^30^, assessing the available evidence (on criteria of geographical and temporal proximity) to distinguish revolt onset dates from dates potentially part of already ongoing revolts. The final cessation dates of revolts are generally ambiguous in comparison to onsets, and are therefore not included in our analysis. For the start and cessation dates of “Syrian Wars” between the Ptolemaic and Seleukid states, we use the compilation by Grainger (Supplementary Note 3)^33^. For dates of priestly decrees, including that written on the famous Rosetta Stone, reflecting accommodations between the kings and the politically powerful assembly of priests, we use the study by Huß^59^, supplemented by a new text from Alexandria (243 BCE)^60^. For land sales, we use the corpus of Egyptian (Demotic) texts assembled by Manning^37^.

### Composite analyses

Composites of social indexes (Fig. 3a–d) are made by averaging 17-year windows of each time series centered on each volcanic eruption with a northern hemisphere forcing of <−1 W/m^2^ ^27^. This window was chosen to correspond to the average interval between volcanic eruptions. It thus represents the longest interval before it becomes probable that a secondary volcanic eruption will occur within the composite window. If a second eruption does occur during a given window, then the values associated with the year of the secondary eruption and the two following years are removed (set to NaN) before compositing. Statistical significance (*P*-values) for the binary time series (internal revolt onset, war cessation, priestly decrees) are calculated using Barnard’s exact test^61^. The 95% confidence threshold for each lag (−1 to −8, 1–8) is also estimated using Barnard’s exact test, accounting for the actual number of observations at each lag after accounting for the masking (noted above) of some non-focal years due to secondary eruptions. Land sale counts are not binary and we thus estimate significance using the 2-sample student’s *t*-test. For our revolt onset dates, we note that the 145 BCE onset date is ambiguous, with 141 BCE representing a potential alternative^30^. However, we find that the frequency of revolt onsets in both eruption years and in the second years following is statistically significant at (at least) the *P* < 0.05 level, regardless of whether we use only the 145 BCE date, the 141 BCE date, both dates (as per Fig. 3a), or neither.

### Volcanic eruption dates and climate forcing

“Eruption years”, in all statistical tests, refer only to those years in which an eruption is estimated to have occurred. All other years are deemed “non-eruption years”. Our “eruption year” dates represent the year in which volcanic sulfate deposition associated with each eruption is first registered in Greenland and Antarctic ice cores, based upon recently refined ice-core chronologies^27^. We consider this the best present estimate for the timing of maximum hemispheric atmospheric sulfuric acid loading (i.e., the year of maximum volcanic forcing) based upon observations of present-day eruptions^62, 63^. For the majority of events, the deposition start year also equates to the year of maximum annual sulfate concentrations in the polar ice cores (e.g., Laki, 1783), though for the largest eruptions, particularly in the tropics (e.g., Tambora 1815, Samalas 1257), maximum annual sulfate deposition may occur one year after the start of deposition on the polar ice-sheets. This can be explained by the longer stratospheric residence times of aerosols from tropical eruptions as compared to high-latitude eruptions^64^. Our forcing estimates represent the time-integrated (i.e., cumulative) volcanic forcing over the full time-span of sulfate deposition for each event, which is at a first order proportional to the NH peak forcing centered at our start dates. Northern Hemispheric (NH) volcanic forcing values are estimated from the global volcanic forcing values reported by^27^, dividing values for tropical eruptions by two, and those from NH extratropical eruptions by one. All volcanic events reported by^27^ for which time-integrated volcanic forcing of the NH falls below a threshold of −1 W/m^2^ (equivalent to approximately one third the strength of Pinatubo 1991 or Krakatoa (Krakatau) 1883, for comparison) are employed in our analyses.

### Sample counts

There were 75 eruption years between the earliest available Nilometer measurement in 622 CE and the construction of the Aswan Low Dam in 1902 CE. Fourteen of these eruptions are excluded from analysis because the eruption dates fall within one of the 190 years where Nile summer flood heights were not measured or have not survived^28^. The two-sample *t*-test comparing the distribution of flood heights between eruption and non-eruption years, and the ranked flood analysis, thus use 61 samples from the eruption-year distribution and 1030 samples from the non-eruption year distribution. One additional eruption was excluded from the composite analysis (Fig. 2c) because it occurred in 626, sufficiently close to the beginning of the Nilometer record that the compositing window falls partially outside the time period of Islamic Nilometer coverage. The mean values and confidence intervals in Fig. 2c are thus calculated, for year 0, using 60 Nile summer flood height values. For other years in Fig. 2c (−8 to −1 and 1–8), the number of samples varies between 44 and 58. These numbers are smaller than 60 because the Nilometer value is missing in the given year, or the data is masked because it is associated in time with a secondary volcanic eruption that occurred within the compositing window. There were 18 eruption years during the 276 year Ptolemaic period (305–30 BCE; Fig. 1b). Of the 96 years during this period for which Nile flood quality evaluations can be made from written records^29^, 8 of these years correspond to eruption years and 88 years do not. The higher relative sampling rate of eruption years in the documentary record, relative to the Ptolemaic period as a whole, may be associated with an increased likelihood of finding written records discussing Nile flood conditions in years where the flood is unusually low. For the composite analyses of social indexes (Fig. 3), we consider all years during the Ptolemaic period, excepting an 8-year buffer at the beginning and end of the period so that no window extends outside the period. This gives us a compositing window of 260 years from 297 BCE to 38 BCE, with 16 eruption years (i.e., omitting eruptions dated to 301 and 299 BCE that fall outside the window). During this 260 year period, 3 of 10 revolt onset dates happened in eruption years, with another 5 occurring within 2 years. Three of 9 Syrian war cessation dates happened in an eruption year, with 2 further occurring 2 years after and 1 occurring 3 years after. Two of nine priestly decrees were issued in eruption years, with 1 additional decree in the first year following an eruption.

### Sensitivity to eruption size

The <−1 W/m^2^ NH cumulative forcing threshold is chosen as a lower-bound indicator of an eruption resulting in a large (and potentially climatically effective) atmospheric injection of sulfate. Our general finding of Nile summer flood height suppression is, however, robust to other threshold choices. Indeed, the magnitude of the observed suppression is larger if a lower cutoff threshold is chosen. For example, if we restrict our analysis to eruptions with a forcing of below 0, −1, −2, −3, −4, or −5 W/m^2^, we observe an average eruption-year decrease in Nile summer flood height of 12, 22, 29, 39, 49, and 61 cm, respectively. These results also act as a general long-period validation of the Islamic Nilometer, and the association between explosive volcanism and Nile summer flood heights, demonstrated here, has diagnostic potential in advancing future efforts to examine the historical basis of the surviving Islamic Nilometer record, a subject meriting further study^51^.

### Nile flood quality

We derive qualitative Nile flood quality indications from D. Bonneau’s compilation of (primarily) Greek papyri^29^, covering 96 years in the Ptolemaic period, 305–30 BCE. Coverage is intermittent because of variable document survival, with particularly high survival for the area of the Fayyum oasis (i.e., a depression or deflation hollow, just west of the Nile and south of Cairo). The papyri variably provide precise measurements of Nile summer flood heights and qualitative assessments of flood extent (e.g., by state officials), augmented by inferences from reported rupturing of dikes and similarly indicative evidence, often attested in multiple documents. We allow a minor dating uncertainty of ±1 year, e.g., arising from multiple co-existing calendars and instances wherein only regnal years are provided, hampering conversion to specific Gregorian years. Following Bonneau, Nile flood quality is ranked on an ordinal scale from 1, “poor” (“mauvaise”) to 8, “strong” (“forte”). See also the treatment of D. Bonneau’s compilation for the post-Ptolemaic era in ref. ^65^.

### Data availability

Data underlying the results published in this article are available accompanying the cited literature, in the Supplementary Information and/or from the authors upon request (ludlowf@tcd.ie).

## Electronic supplementary material

Supplementary Information
