# Supplementary material for: Volcanic suppression of Nile summer flooding triggers revolt and constrains interstate conflict in ancient Egypt
Source: Nat Commun. 2017 Oct 17;8:900. doi: 10.1038/s41467-017-00957-y (PMC5645420; doi:10.1038/s41467-017-00957-y)
Supplement: Supplementary file 1 — Supplementary Information [file 41467_2017_957_MOESM1_ESM.pdf]

Supplementary Table 1: Models used in the CMIP5 analysis

**Model name**	**Number of integrations**
ACCESS1.0	1
ACCESS1.3	1
BCC-CSM1	3
BCC-CSM1-1-m	3
BNU-ESM	1
CanESM2	5
CCSM4	6
CESM1 (BGC)	1
CESM (CAM5)	3
CNRM-CM5	10
CSIRO-Mk3.6.0	10
FGOALS-s2	3
GFDL-CM3	5
GISS-E2-H	5
GISS-E2-R	6
HadGEM2-ES	4
MIROC5	4
MPI-ESM-LR	3
MPI-ESM-MR	3
MRI-CGCM3	3
NorESM1-M	3

This is the combination of the set of models used by Iles and Hegerl (2014) and Driscoll *et al.* (2012) in analyses of the Coupled Model Intercomparison Project 5 (CMIP5) response to volcanic eruptions, and details on model resolution and the applied volcanic forcing can be found in those papers. See Iles, C. E., & Hegerl, G. C. The global precipitation response to volcanic eruptions in the CMIP5 models. *Env. Res. Lett.* 9, 104012 (2014), and Driscoll, S., Bozzo, A., Gray, L. J., Robock, A. and Stenchikov, G. Coupled Model Intercomparison Project 5 (CMIP5) simulations of climate following volcanic eruptions, *J. Geophys. Res.* 117, D17105 (2012).

Supplementary Table 2. Internal revolt onset in Ptolemaic Egypt

**Onset dates, 305-30 BCE**	**Location**
245 BCE	Likely extensive
217 BCE	Revolt of demobilized soldiers after Raphia
207 BCE	Throughout Egypt
168 BCE	Throughout Egypt
156 BCE	Pathyris (Upper Egypt)
145 BCE	Edfu (Upper Egypt)
141 BCE*	Edfu (Upper Egypt)
131 BCE	Throughout Egypt
107 BCE	Upper Egypt
84 BCE**	Middle Egypt

Source: Adapted (see Methods) from Anne-Emmanuelle Veïsse, *Les “Revoltes Égyptiennes”: Recherches sur les troubles intérieurs en Égypte du règne Ptolémée III à la conquete Romaine* (Peeters Publishers, 2004, pp. 78-79).

* Variant onset date of 145 BCE revolt.

** Revolt uncertainly dated to the 33rd or 34th regnal year of Ptolemy IX Soter II; we thus take 84 BCE as *terminus post quem* for this event.

Supplementary Table 3. The Syrian Wars

Wars	Ruler & reign	Start & cessation, BCE
1st	Ptolemy II (r. 285–246 BCE)	274-271
2nd	Ptolemy II	260-253
3rd	Ptolemy III (r. 246–222 BCE)	246-241
4th	Ptolemy IV (r. 221–204 BCE)	219-217
5th	Ptolemy V (r. 204–181 BCE)	202-195
6th	Ptolemy VI (r. 186–145 BCE)	170-168
7th	Ptolemy VI	147-145
8th	Ptolemy VIII (r. 144-132; 126-116 BCE)	128-123
9th	Ptolemy X (r. 110/109, 107-88 BCE)	103-96

Source: Grainger, J. D. *The Syrian Wars* (Brill, 2010).
Note: “r.” in the middle column stands for “reign”, i.e., of each king.

Supplementary Table 4. Priestly decrees in Ptolemaic Egypt

Decree dates 305-30 BCE	Location issued, where known
243	Alexandria
238	Canopus
217	Raphia
196	Memphis (aka ‘Rosetta Stone’)
186	Decree of 186
184	Decree of 184
182	Decree of 182
161	Decree of 161
39	Kallimachos

Sources: Adapted (see Methods) from Huß, W. Die in ptolemaiischer Zeit verfaßten Synodal-Dekrete der ägyptischen Priester. *Zeitschrift für Papyrologie und Epigraphik* 88, 189-208 (1991), and El-Masry, Y., Altenmüller, H. & Thissen, H. J., *Das Synodaldekret von Alexandria aus dem Jahre 243 v. Chr.* (Beck, 2012).
Note: This table includes only those priestly decrees of presently secure date.

Supplementary Note 1.
Papyrus Edfu 8. A text originating from Edfu (i.e., Apollinopolis-the-Great) dated to the middle of the 3rd century BCE. See A. Lukaszewicz, "Le Papyrus Edfou 8 soixante ans après," in *Tell-Edfou soixante ans après: Actes du colloque franco-polonais, Le Caire- 15 Octobre 1996*. Cairo: Institut Français d'Archéologie Orientale, 1999, pp. 31-32. This petition, written in Greek, is probably a draft and details the impacts of Nile failure in this period, also illustrating attempts to mitigate this through technological innovation. The text, requesting a royal audience, was written by a soldier (as indicated by his name and title, kleruch, a type of reserve soldier) living in Edfu, a major temple town in Upper (i.e., southern) Egypt. The text is not dated specifically, but is generally thought (based on the text's palaeography and description of severe flood failure of three years' duration) to have been written in the middle of the third century BCE.

To King Ptolemy, Greetings, from Philotas, the fire-signaller, one of the kleruchs in Apollinopolis-the-Great. Given that now and for a long time, the inundation has become insufficient, I want, O King, to inform you of a certain machine the use of which does no damage and by means of which the country may be saved. Since during the last 3 years the river has not flooded, the dryness will produce a famine that […] but if you wish, this will be a year of good flood. I ask you, O King, if it seems good to you, to order Ariston the strategos, to grant me 30 days sustenance, and to send for me as quickly as possible to you or […] a petition so that, if it pleases you, seed will grow immediately. Thanks to your decision, within 50 days there will immediately follow a plentiful harvest throughout the whole Thebaid [i.e., a major administrative district in Upper Egypt]. Farewell.

Supplementary Note 2.
Canopus Decree (*OGIS* 56), 4 March 238 BCE. This is a partial translation of the Greek version of the trilingual priestly decree. Translation in M.M. Austin, *The Hellenistic world from Alexander to the Roman conquest: A selection of Ancient sources in translation*. 2nd ed. Cambridge: Cambridge University Press, 2006, p.471. For a full treatment of the text, see S. Pfeiffer, *Das dekret von Kanopos (238 v. Chr.): Kommentar und historische auswertung eines dreisprachigen synodaldekretes der ägyptischen priester zu Ehren Ptolemaios' III. und seiner familie*. München: K.G. Saur, 2004. On the location of the "many other places" referred to in the text, see K. Buraselis, Ptolemaic grain, seaways and power, In: *The Ptolemies, the sea and the Nile: Studies in waterbourne power*, Ed. K. Buraselis, M. Stefanou, and D.J. Thompson. Cambridge: Cambridge University Press, 2013, p.101. In this text, Ptolemy III is portrayed as having saved Egypt during a recent Nile failure (likely in the 240's BCE), at great personal expense by importing grain from external rainfed (i.e., Nile-independent) territories, an example of state-level coping strategies.

…. and when on one occasion the rise of the river [i.e., the Nile] was insufficient and all the inhabitants of the country were terrified at what had happened and remembered the disaster that occurred under some of the previous kings, under whom it happened that all the people living in the land suffered from a drought, they showed their care for the residents in the temples and the other inhabitants of the country, and showed much foresight and sacrificed a large part of their revenues for the salvation of the population, and by importing corn into the country from Syria, Phoenicia and Cyprus and many other places at great expense, they saved the inhabitants of Egypt….

Supplementary Note 3.
Josephus, *Against Apion* 2.60. Translation by John M.G. Barclay, *Flavius Josephus: Against Apion. Translation and commentary.* Leiden: Brill, 2007, p. 202. This text, a lengthy and complex work concerning the history of Judaism in the Mediterranean world, likely written in the second century CE, indirectly references Cleopatra's opening of royal granaries to feed a starving population in during famine. The capture of Alexandria by Caesar refers to Octavian's successful invasion of Egypt in 30 BCE. The reference to famine here credibly refers back to

the events in the late 40's BCE, when other historical sources indicate severe Nile failure. Josephus claims in this excerpt that Cleopatra had not allowed the Jews at Alexandria emergency grain rations during the famine because they were not citizens, though this allegation is unconfirmed (see: Joyce Tyldesley, *Cleopatra: Last queen of Egypt*. London: Profile, 2008, p. 141).

Finally, when Alexandria had been captured by Caesar, she was reduced to such straits that she judged she could hope for survival if she could kill the Judeans with her own hands, having been conspicuous for her cruelty and disloyalty to everyone. Would you not think it something to be proud of if, as Apion says, she did not distribute grain rations to the Judeans at a time of famine?

Supplementary Note 4.
Justin, *Epitome* 27.1.19. Translation by John S. Watson at 'Corpus scriptorium Latinorum: A digital library of Latin literature', http://www.forumromanum.org/literature/justin/english/trans27.html (accessed 05 August 2017). This text references the recall of Ptolemy III from his successful campaign against the Seleukid Empire during the Third Syrian War to face "disturbances at home", during the 240s BCE.

But Berenice, before succour could arrive, was surprised by treachery, as she could not be taken by force, and killed. The deed was regarded by everyone as an atrocity; and all the cities, in consequence, which had revolted (after having equipped a vast fleet), being suddenly alarmed at this instance of cruelty, and wishing to take revenge for her whom they had meant to defend, gave themselves up to Ptolemy, who, if he had not been recalled to Egypt by disturbances at home, would have made himself master of all Seleucus's dominions.

Supplementary Note 5.
Ptolemy III Chronicle. Excerpt of translation by R. J. van der Spek & I. Finkel of British Museum cuneiform tablet 34428, detailing Ptolemy III's arrival at Babylon during the Third Syrian War, available at 'Ptolemy III Chronicle (BCHP 11)', http://www.livius.org/cg-cm/chronicles/bchp-ptolemy_iii/bchp_ptolemy_iii_01.html (accessed 05 August 2017).

Kislîmu [IX = 26 November - 25 December 246 BCE]

[..........] …. [..........]

[.....Ptolemy the k]ing of Egypt arrived at [Seleukia, the royal city, which is on the] Euphrates[sic] and the Royal Cana[l]. [The chief guardian who is in the palace[[sh]ut in [the army of the ki]ng, which was in Babylon, before P[tolemy?] The gates [...........] he captured and locked. Tebêtu [X = 26 December 246-23 January 245 BCE]. That month, day 15 [t]h, (9 January), the Hanaean troops, who did not fear the gods, who were clad in iron panoply, transferred battle equipment [and] numerous [siege en]gines, from the city of Seleucia, the royal city, which is on the Euphrates[sic], to Babylon. Day 19th (13 January) they did battle with the commander of the Bêlet-Ninua Citadel. The people who were in the citadel, became frightened and they went out from the citadel. They arrived at the palace of the king. That day, the people were slaughtered with iron weapons [b]y the Hanaean troops. That month, the 24th day (18 January), a certain renowned prince, a representative of the king, who from the land of Egypt had come (= Xanthippus[?]), with troops in great numbers, who were clad in iron panoply, from Seleucia, the royal city, which is on the Euphrates[sic], arrived at Babylon. On the 26th day (20 January) into Esagila……….

Supplementary Note 6.

***SB* XXIV 1597.** A Greek papyrus at Trinity College Dublin, Pap. Gr. 274, dated c.186 BCE, during the extensive Theban revolt, written by a royal official reporting on tax collection difficulties and efforts to restore the land to taxable production. Translation by W. Clarysse, 'The great revolt of the Egyptians (205-186 BCE)', 2004, http://www.lib.berkeley.edu/sites/default/files/files/TheGreatRevoltoftheEgyptians.pdf (accessed 05 August 2017). See also B. C. McGing, Revolt Egyptian style: Internal opposition to Ptolemaic rule, *Archiv für Papyrusforschung* 43, 1997, pp. 299-310.

… From the time of the revolt of Chaonnophris it happened that most of the farmers were killed and the land has gone dry. When, therefore, as is customary, the land which did not have owners was registered among the "ownerless land," some of the survivors encroached upon the land bordering their own and got hold of more than was allowed. Their names are unknown since nobody pays taxes for this land to the treasury. But of the cultivated area nothing has been overlooked, because the land–measurement of what is sown has taken place each year, and the taxes are being executed – –

Supplementary Note 7.

Excerpts of the Kallimachos Decree (*OGIS* 194), March, 39 BCE. Translation by S.M. Burstein, *The Hellenistic Age from the battle of Ipsos to the death of Kleopatra VII.* Cambridge: Cambridge University Press, 1985. Text 111. This text details the impacts of famine arising from implied Nile failure in the 40s BCE, continuing in 39 BCE, the date of this decree, as well as relief efforts.

……Kallimachos, the kinsman [and strategos and] revenue officer for the district of Thebes, and gymnasiarch and cavalry-commander, previously having taken over the city, which had been ruined [as a result of] manifold [disastrous] circumstances, tended it carefully [and maintained it] unburdened [in] complete peace……..the severe famine caused by a crop failure like none hitherto recorded, and when the city had been almost crushed by [need] ...he voluntarily contributed to the salvation of each of the local inhabitants… ...The famine, however, continued in the present year and became even worse and [...] [...] a failure of the flood and misery far worse than ever before reigning throughout the whole [land] and the condition of the city being wholly critical….

Supplementary Note 8.

Seneca, *Natural Questions*, IVa.2.16.

Translation by T.H. Corcoran, *Seneca: Natural Questions, Books 4-7*. Cambridge: Harvard University Press, 1972, p. 33. This excerpt from Seneca's *Natural Questions*, written in the first century CE, notes poor Nile flooding during Cleopatra's reign. The tenth and the eleventh years of Cleopatra's reign correspond to 43-41 BCE. The "Callimachus" referred to is thought to be the famous Alexandrian poet (d. 240 BCE), and the nine years Nile failure described here must pre-date 240 BCE, but is presently difficult to date more accurately. The reference is thought to derive from Callimachus's now-lost treatise *On Rivers* (see: Jonathan Tracy, *Lucan's Egyptian civil war*. Cambridge: Cambridge University Press, 2014, p.168). Such works highlight the importance of Nile variability in the consciousness of ancient scholarship.

It is well established that in the reign of Cleopatra the Nile did not flood for two successive years, the tenth and eleventh of her reign. They say that this was a sign of the loss of power for the two rulers of the world, for the empire of Antony and Cleopatra did fall. Callimachus is my authority that in earlier times the Nile did not flood for nine years.

Supplementary Note 9.

Pliny, *Natural History* 5.10.58. This excerpt from Pliny's famous *Natural History*, written in the first century CE, describes the agricultural consequences of insufficient Nile summer flooding, also indicating how an apparently small height difference was deemed to produce large societal impacts. Translation at The Perseus Project, http://www.perseus.tufts.edu/hopper/text?doc=Plin.+Nat.+5.10&fromdoc=Perseus%3Atext%3A1999.02.0137 (accessed 05 August 2017).

Its most desirable height is sixteen cubits; if the waters do not attain that height, the overflow is not universal; but if they exceed that measure, by their slowness in receding they tend to retard the process of cultivation. In the latter case the time for sowing is lost, in consequence of the moisture of the soil; in the former, the ground is so parched that the seed-time comes to no purpose. The country has reason to make careful note of either extreme. When the water rises to only twelve cubits, it experiences the horrors of famine; when it attains thirteen, hunger is still the result; a rise of fourteen cubits is productive of gladness; a rise of fifteen sets all anxieties at rest; while an increase of sixteen is productive of unbounded transports of joy. The greatest increase known, up to the present time, is that of eighteen cubits, which took place in the time of the Emperor Claudius; the smallest rise was that of five, in the year of the battle of Pharsalia [i.e., 48 BCE], the river by this prodigy testifying its horror, as it were, at the murder of Pompeius Magnus.
